# The Correlation of Endoscopic Findings and Clinical Features in Korean Patients with Scrub Typhus: A Cohort Study

**DOI:** 10.1371/journal.pone.0155810

**Published:** 2016-05-19

**Authors:** Jun Lee, Dong-Min Kim, Na Ra Yun, Young Dae Kim, Chan Guk Park, Man Woo Kim

**Affiliations:** Department of Internal Medicine, School of Medicine, Chosun University, Gwangju, Republic of Korea; University Hospital Llandough, UNITED KINGDOM

## Abstract

Scrub typhus is an infectious disease caused by *Orientia tsutsugamushi*-induced systemic vasculitis, but the involvement of the gastrointestinal tract and the endoscopic findings associated with scrub typhus are not well understood. We performed a prospective study and recommend performing esophagogastroduodenoscopy (EGD) for all possible scrub typhus patients, regardless of gastrointestinal symptoms. Gastrointestinal symptoms, endoscopic findings and clinical severity based on organ involvement and ICU admission were analyzed. Gastrointestinal symptoms occurred in up to 76.4% of scrub typhus patients. The major endoscopic findings were ulcers (43/127, 33.9%). Interestingly, 7.1% (9/127) of the patients presented with esophageal candidiasis. There was no correlation between the presence or absence of gastrointestinal symptoms and the endoscopic grade (P = 0.995). However, there was a positive correlation between the clinical severity and the endoscopic findings (P = 0.001). Sixty-three percent of the patients presented with erosion or ulcers on prospectively performed endoscopic evaluations, irrespective of gastrointestinal symptoms. Gastrointestinal symptoms did not reflect the need for endoscopy. Scrub typhus patients could have significant endoscopic abnormalities even in the absence of gastrointestinal symptoms.

## Introduction

Scrub typhus is endemic in Korea, Japan, Southeast Asia, the western Pacific Islands, Australia, China, south-central Russia, India, and Sri Lanka [1–3]. More than 1 million cases occur annually worldwide [4]. The number of scrub typhus patients has increased in South Korea, and 69,210 scrub typhus cases were reported in South Korea from 2001–2013 [5].

Scrub typhus is an acute febrile illness caused by *Orientia tsutsugamushi*-induced systemic vasculitis, which can involve the lungs, heart, liver, skin, central nervous system, and gastrointestinal tract. Scrub typhus can achieve recovery almost without sequelae through early treatment, such as administering effective antibiotics. In some cases, however, severe complications such as intestinal pneumonia, acute respiratory distress syndrome, acute renal failure, and multiple organ dysfunction occur, which may lead to death [6]. Gastrointestinal manifestations are very rarely reported for scrub typhus. In septic patients with scrub typhus, gastrointestinal symptoms such as vomiting, nausea, diarrhea, and hematemesis or melena can occur [7]. However, the endoscopic findings associated with gastrointestinal vasculitis are not well known. In one previous study conducted in South Korea, the major endoscopic features of scrub typhus were superficial mucosal hemorrhage, multiple erosions, and ulcers with no predilection for particular sites, as well as unusual vascular bleeding [8]. However, this previous study had a major limitation: it enrolled only scrub typhus patients with gastrointestinal symptoms. In our study, we prospectively performed endoscopy in all patients, regardless of gastrointestinal symptoms. We investigated upper gastrointestinal tract involvement and characteristic endoscopic findings in scrub typhus patients, and we determined the correlation between the extent of endoscopic lesions and clinical severity and the correlation between the extent of endoscopic lesions and the presence or absence of gastrointestinal symptoms.

## Materials and Methods

**Fig 1:**
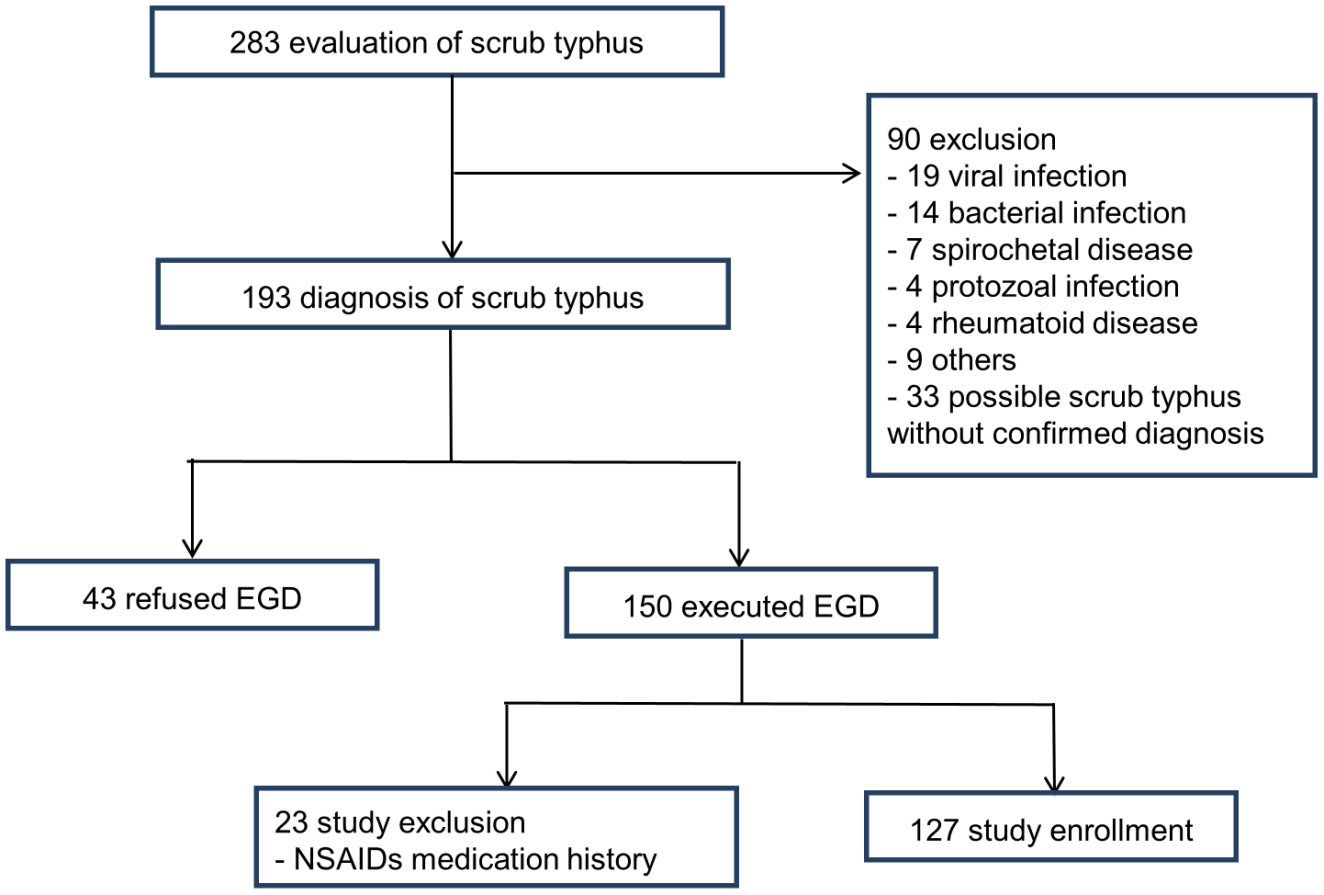
Patient data for the study on the endoscopic findings of scrub typhus.

From September 2006 to December 2008, 283 patients visited Chosun University Hospital with a chief complaint of fever or rash. Scrub typhus was diagnosed when a four-fold or greater increase in IgM or IgG antibody titers, measured by indirect immunofluorescent assay, was observed in the acute and convalescent stages or when PCR was positive, as described previously with slight modification [9]. Of these patients, 90 did not meet the diagnostic criteria for scrub typhus (19 cases of other viral infections, such as hemorrhagic fever with renal syndrome [HFRS], hepatitis or influenza; 14 cases of bacterial infections; seven cases of spirochetal disease, such as leptospirosis; four cases of protozoal infections, such as malaria; four cases of rheumatoid diseases, such as systemic lupus erythematosus or adult-onset Still’s disease; nine cases of other illnesses; and 33 cases of possible scrub typhus without confirmed diagnoses). The diagnosis of scrub typhus was confirmed in 193 patients. Patients with possible scrub typhus were asked to enroll in the study, and 150 patients underwent an endoscopy (43 patients refused endoscopic examinations) using a video endoscope (GIF-Q260, Olympus Co, Tokyo, Japan) within three days after admission. We excluded 23 of these patients because they had a medical history of taking non-steroidal anti-inflammatory drugs (NSAIDs); thus, 127 patients were included, and their medical and endoscopic records were analyzed. (Fig 1) To minimize the inter-observer variation, two endoscopists (authors) reviewed the endoscopic records. The study was approved by the institutional review board of Chosun University Hospital. All participants provided their written informed consent to participate in our study.

The endoscopic findings were graded from I to IV as follows: grade I: normal findings; grade II: mucosal hyperemia; grade III: mucosal erosion with or without superficial hemorrhage; and grade IV: mucosal ulceration with or without active bleeding [8].

We classified clinical severity into three grades (mild, moderate, and severe) according to the following six clinical indicators: admission to the intensive care unit (ICU); fever; thrombocytopenia (<15,000/μL); hepatic involvement (elevated [>40 U/L] serum levels of alanine aminotransferase [ALT]); respiratory involvement (arterial PO_2_<70 mm Hg), renal involvement (elevated [>1.5 mg/dL] serum creatinine); and neurologic involvement (confusion). Severe cases were defined as the involvement of more than five organ systems or admission to the ICU. Moderate cases were defined as the involvement of three or four organ systems, and mild cases were defined as the involvement of two or fewer organ systems, as described previously with slight modification [10].

We evaluated the gastrointestinal symptoms and the endoscopic grade to determine the correlation between clinical severity and endoscopic findings and to ascertain the necessity for endoscopy regardless of symptoms.

### Statistical analysis

The statistical evaluation was performed using SPSS software (version 18.0 Chicago, IL). To compare qualitative variables, the chi-squared test was used. To assess the correlation between endoscopic findings and gastrointestinal symptoms, Pearson’s correlation coefficient was calculated.

## Results

### Demographic and clinical characteristics

The demographic data and clinical features of the patients with scrub typhus are presented in Table 1. We enrolled 127 patients with scrub typhus (81 women, 46 men). The mean age of the patients was 61.67 years old. The average time from symptom onset to admission was 6.9 days. The average length of hospitalization was 7.5 days.

**Table 1:** Demographic and clinical characteristics of scrub typhus.

Characteristic	Value. n = 127 (n [%])
Age, y, mean(SD)	61.67 (13.63)
Sex, no. (%)	
Male	46 (36.2)
Female	81 (63.8)
Duration, days, mean (SD)	
Symptom onset to hospitalization	6.9 (7.705)
Hospitalization days	7.5 (4.417)
Clinical characteristics, no. (%)	
Headache	109 (85.8)
Chills	104 (81.9)
Myalgia	86 (67.7)
Cough	40 (31.5)
Fever	92 (73.2)
Eschar	123 (96.9)
Rash	112 (88.2)
Underlying disease, no. (%)	
Hypertension	17 (13.4)
Diabetes	7 (5.5)
Cardiovascular disease	1 (0.8)
Lung disease	2 (1.6)
Malignancy	4 (3.1)

SD, standard deviation

### Endoscopic features

**Fig 2:**
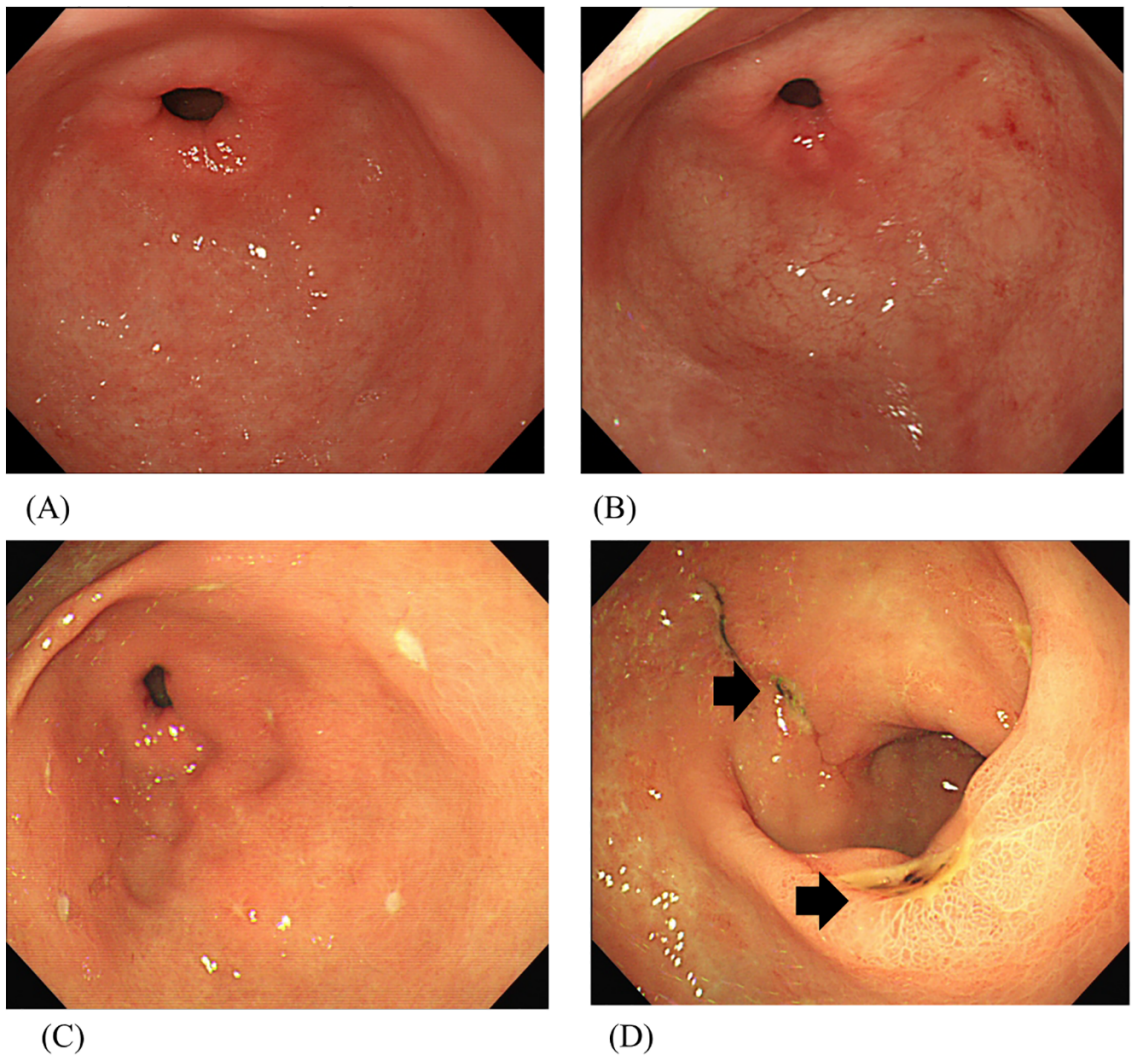
Endoscopic findings of scrub typhus. (A) Grade I: normal. (B) Grade II: mucosal erythema. (C) Grade III: mucosal erosion. (D) Grade IV: ulcer; black arrow and/or active bleeding.

The endoscopic findings that can develop in scrub typhus are erythema, erosion, and ulcers. Grade I features were present in 26 patients (20.5%), grade II in 21 patients (16.5%), grade III in 37 patients (29.1%), and grade IV in 43 patients (33.9%). (Fig 2) The major findings were ulcers (43/127, 33.9%); 56% of the patients who had ulcers (24/43) had multiple ulcers, with an average of 3.5 ulcers per patient. In the patients with multiple ulcers, the site of predilection of the ulcer was the antrum (67%) or the duodenum (20%). Nineteen patients who had single ulcers had multiple erosions, excluding two patients. In the patients with a single ulcer, the site of predilection the ulcers was the duodenum (47%) or antrum (37%). (Table 2) Active bleeding was observed in five patients (3.9%). Four of these patients were managed with endoscopic hemostasis, but one patient expired from hemorrhage-induced disseminated intravascular coagulation. Epinephrine injection and argon plasma coagulation (APC) were administered in cases of endoscopic hemostasis. Esophageal candidiasis was observed in 9 patients (7.1%). Esophageal candidiasis was endoscopically diagnosed in the presence of typical sparse or coalescent white plates covering the esophageal mucosa. The diagnosis was always confirmed with esophageal brushing or biopsy.

**Table 2:** Location of ulcers in scrub typhus patients.

Location	Single ulcer (n = 19)	Multiple ulcers (n = 85/24 patients; average ulcer count = 3.5)
Duodenum	9 (47%)	17 (20%)
Stomach	10 (53%)	68 (80%)
antrum	7	57
body	3	9
fundus	0	0
cardia	0	2

### Gastrointestinal manifestations and severity

The frequency of gastrointestinal symptoms was as follows: dyspepsia (49.6%), nausea (44.1%), abdominal pain (23.6%), vomiting (14.2%), and melena or hematemesis (7.1%). (Table 3) Gastrointestinal symptoms occurred in up to 73.2% (93/127) of the scrub typhus patients in our study. The endoscopic grades of the patients with gastrointestinal symptoms were as follows: grade I: 19 patients; II: 15 patients; III: 27 patients; and IV: 32 patients. The endoscopic grades of the patients without gastrointestinal symptoms were as follows: grade I: seven patients; II: six patients; III: 10 patients; and IV: 11 patients. (Table 4) There was no correlation between the presence or absence of gastrointestinal symptoms and endoscopic grade. Regarding clinical severity, 65 patients were classified as mild, 50 as moderate, and 12 as severe. The severe patients were further classified as follows: endoscopic grade I (2/12), grade II (0/12), grade III (3/12), and grade IV (7/12; Table 5). There was a positive correlation between clinical severity and endoscopic findings (Pearson’s coefficient 0.282, P = 0.001).

**Table 3:** Gastrointestinal symptoms of scrub typhus.

Symptoms	No. of total patients (n = 127)	No. of patients with endoscopic grade III or IV (n = 80)	No. of patients with endoscopic grade I or II (n = 47)	P-value
Dyspepsia	63 (49.6%)	43 (53.8%)	20 (42.6%)	0.223
Nausea	56 (44.1%)	33 (41.3%)	23 (48.9%)	0.400
Abdominal pain	30 (23.6%)	20 (25.0%)	10 (21.3%)	0.633
Vomiting	18 (14.2%)	14 (17.5%)	4 (0.09%)	0.161
Melena or hematemesis	9 (7.1%)	8 (10%)	1 (0.02%)	0.095
No symptoms	34 (26.8%)	21 (26.3%)	13 (27.7%)	0.995

**Table 4:** Endoscopic findings according to gastrointestinal symptoms.

Grade	No. of patients (n = 127)	
	Gastrointestinal symptom (+) (n = 93)	Gastrointestinal symptom (-) (n = 34)
I Normal	19 (20.4%)	7 (20.6%)
II Mucosal hyperemia	15 (16.2%)	6 (17.6%)
III Mucosal erosion	27 (29.0%)	10 (29.4%)
IV Ulceration or active bleeding	32 (34.4%)	11 (32.4%)
Total	93 (100%)	34 (100%)

Pearson’s coefficient = 0.067, P = 0.995

**Table 5:** The correlation between the grading of endoscopic lesions and the clinical severity of scrub typhus.

Grade	Clinical severity. No. of patients (n = 127)		
	Mild	Moderate	Severe
I Normal	19	5	2
II Mucosal hyperemia	13	8	0
III Mucosal erosion	18	16	3
IV Ulceration or active bleeding	15	21	7

Pearson’s coefficient 0.282, P = 0.001

## Discussion

The present study shows that gastrointestinal symptoms developed frequently in scrub typhus, and the major endoscopic findings were erosion and ulcers. Although the precise mechanism of gastrointestinal involvement associated with scrub typhus is not yet known, it can be speculated that direct involvement of systemic vasculitis leads to gastrointestinal symptoms and signs. It has been speculated that when there is gastrointestinal involvement in systemic vasculitis, a biopsy of the superficial mucosal erosion in Henoch-Schonlein purpura (HSP) patients could confirm vasculitis of the small vessels associated with the deposition of immunoglobulin A [11]. However, the gastrointestinal symptoms and endoscopic findings are significantly different between HSP and scrub typhus. Gastrointestinal symptoms occur in up to 85% of HSP patients, and they manifest as severe problems, such as intussusception, obstruction, and perforation [12–15]. In the reports by Aung-Thu et al, the gastrointestinal symptoms of scrub typhus patients were vomiting (65%), nausea (60%), diarrhea (45%), melena or hematemesis (25%) [7]. These gastrointestinal symptoms are consistent with those found in the present study, but the frequency of the symptoms was different. In our study, the frequency of gastrointestinal symptoms in scrub typhus patients was as follows: dyspepsia (49.6%), nausea (44.1%), abdominal pain (2.6%), vomiting (14.2%), and melena or hematemesis (7.1%). The reason for the difference is that the Aung et al study enrolled small number of septic patients with scrub typhus (n = 20). Gastrointestinal symptoms occurred in up to 76.4% of scrub typhus patients but usually did not manifest as severe problems, as in HSP. The characteristic endoscopic findings of HSP are erythema, petechiae, and hemorrhagic erosion [16,17]. The duodenum and the small intestine are the most frequently involved sites [11,18]. In our study, the major endoscopic findings observed in scrub typhus were erythema, erosion, and ulcers (79.5%). The preferred sites were the antrum and the duodenal bulb. For this reason, the distinction between the systemic vasculitis of scrub typhus and other types of vasculitis must be considered. A previous Korean study reported erosion and ulcers manifesting in any preferred site in scrub typhus patients [8]. Our data showed that there were predominant sites, including the antrum and duodenal bulb. This discrepancy could have occurred because our data were prospective and our study had a larger sample size than the previous study.

Our study excluded 23 patients who had a medical history of taking NSAIDs because NSAIDs are an independent cause of ulcers. When we analyzed the scrub typhus patients who had a medical history of taking NSAIDs, gastrointestinal symptoms occurred in up to 56.5% (13/23). Interestingly, patients who had a medical history of taking NSAIDs tended to have lower rate of gastrointestinal symptoms (56.5% vs 73.2%), but the difference was not statistically significant (P = 0.105). At least, the scrub typhus patients who had a medical history of taking NSAIDs did not complain more of gastrointestinal symptoms compared with the patients who did not take NSAIDs.

We recommend esophagogastroduodenoscopy for all patients, regardless of gastrointestinal symptoms. There was no correlation between the presence or absence of gastrointestinal symptoms and the endoscopic grade (P = 0.995). This discrepancy might have occurred because the gastrointestinal symptoms of scrub typhus patients tended to be hidden by other systemic symptoms, such as fever, chills and headache. The absence of gastrointestinal symptoms may not rule out gastrointestinal involvement in scrub typhus.

We did find a positive correlation between clinical severity and the endoscopic findings (Pearson’s coefficient 0.282, P = 0.001). Esophagogastroduodenoscopy is an effective procedure for the early determination of clinical complication severity.

In a Korean population that underwent upper endoscopy during a health check-up, the prevalence of esophageal candidiasis was 0.3%, constituting a significant increase [19]. Interestingly, 7.1% (9/127) of the patients in our study were diagnosed with esophageal candidiasis. Mucocutaneous candida infections are commonly associated with a defective cell-mediated immune response that involves the subnormal production of lymphokines by T-cells in response to candida antigens [20,21]. A decrease in lymphocytes in scrub typhus patients has been reported [22]. This decrease was mainly related to a significant reduction in CD4+ cells during the acute phase, which could contribute to develop esophageal candidiasis [22]. However, only one out of 9 patients with esophageal candidiasis showed a decrease in lymphocytes (WBC: 3,700 mm^3^, lymphocyte 18.5%). A possible explanation for this discrepancy is differences in sampling times and in the development of esophageal candidiasis. Therefore, further studies are needed to determine the exact mechanism.

This study had a few limitations. First, it was a single-center study in a local area. Second, the patients enrolled in the study were admitted to a tertiary medical center; therefore, the possibility of overexpression was high. Third, in South Korea, because of the low fee for endoscopy and the high incidence of gastric cancer, individuals older than 40 years old can elect to receive endoscopy regardless of whether they present warning signs or symptoms. For other countries, cost-efficacy studies are needed. Fourth, we did not completely determine the participants’ past medical history of using anti-ulcer medications (histamine receptor antagonists, sucralfate, etc.) that can be purchased over the counter. Fifth, we did not check for *Helicobacter pylori* infection, which is a major cause of ulcer. This study emphasized two points. First, it is the first prospective study to recommend EGD for all possible scrub typhus patients. Second, our study was the first to present an association between esophageal candidiasis and scrub typhus.

## Conclusion

In summary, gastrointestinal symptoms occurred in up to 76.4% of scrub typhus patients. The major endoscopic findings in scrub typhus were erosion and ulcers. Our study suggests that the presence or absence of gastrointestinal symptoms does not reflect the need for endoscopy; however, further, more completely planned systematic studies are needed. Our study is the first report to present an association between esophageal candidiasis and scrub typhus.

## References

[1] Kelly DJ, Fuerst PA, Ching WM, Richards AL. Scrub typhus: the geographic distribution of phenotypic and genotypic variants of Orientia tsutsugamushi. Clin Infect Dis. 2009;48 Suppl 3: S203–230. 10.1086/596576 19220144

[2] Li T, Yang Z, Dong Z, Wang M. Meteorological factors and risk of scrub typhus in Guangzhou, southern China, 2006–2012. BMC Infect Dis. 2014;14: 139 10.1186/1471-2334-14-139 24620733 PMC3995673

[3] Kelly DJ, Foley DH, Richards AL. A spatiotemporal database to track human scrub typhus using the VectorMap application. PLoS Negl Trop Dis. 2015;9: e0004161 10.1371/journal.pntd.0004161 26678263 PMC4683066

[4] Watt G, Parola P. Scrub typhus and tropical rickettsioses. Curr Opin Infect Dis. 2003;16: 429–436. 10.1097/01.qco.0000092814.64370.70 14501995

[5] Jeung YS, Kim CM, Yun NR, Kim SW, Han MA, Kim DM. Effect of latitude and seasonal variation on scrub typhus, South Korea, 2001–2013. Am J Trop Med Hyg. 2016;94: 22–25. 10.4269/ajtmh.15-0474 26503283 PMC4710434

[6] Cracco C, Delafosse C, Baril L, Lefort Y, Morelot C, Derenne JP, et al Multiple organ failure complicating probable scrub typhus. Clin Infect Dis. 2000;31: 191–192. 10.1086/313906 10913423

[7] Aung T, Supanaranond W, Phumiratanaprapin W, Phonrat B, Chinprasatsak S, Ratanajaratroj N. Gastrointestinal manifestations of septic patients with scrub typhus in Maharat Nakhon Ratchasima Hospital. Southeast Asian J Trop Med Public Health. 2004;35: 845–851. 15916079

[8] Kim SJ, Chung IK, Chung IS, Song DH, Park SH, Kim HS, et al The clinical significance of upper gastrointestinal endoscopy in gastrointestinal vasculitis related to scrub typhus. Endoscopy. 2000;32: 950–955. 10.1055/s-2000-9621 11147943

[9] Kim DM, Yun NR, Yang TY, Lee JH, Yang JT, Shim SK, et al Usefulness of nested PCR for the diagnosis of scrub typhus in clinical practice: a prospective study. Am J Trop Med Hyg. 2006;75: 542–545. 16968938

[10] de Sousa R, Ismail N, Nobrega SD, Franca A, Amaro M, Anes M, et al Intralesional expression of mRNA of interferon- gamma, tumor necrosis factor- alpha, interleukin-10, nitric oxide synthase, indoleamine-2,3-dioxygenase, and RANTES is a major immune effector in Mediterranean spotted fever rickettsiosis. J Infect Dis. 2007;196: 770–781. 10.1086/519739 17674321

[11] Kato S, Shibuya H, Naganuma H, Nakagawa H. Gastrointestinal endoscopy in Henoch-Schonlein purpura. Eur J Pediatr. 1992;151: 482–484. 1396906 10.1007/BF01957748

[12] Szer IS. Henoch-Schonlein purpura: when and how to treat. J Rheumatol. 1996;23: 1661–1665. 8877944

[13] Martinez-Frontanilla LA, Haase GM, Ernster JA, Bailey WC. Surgical complications in Henoch-Schonlein Purpura. J Pediatr Surg. 1984;19: 434–436. 6481588 10.1016/s0022-3468(84)80269-9

[14] Clark CV, Hunter JA. Anaphylactoid purpura presenting as a medical and surgical emergency. Br Med J (Clin Res Ed). 1983;287: 22–23. 10.1136/bmj.287.6384.22 PMC1548144 6407680

[15] Okano M, Suzuki T, Takayasu H, Harasawa S, Sato T, Tsutsumi Y, et al Anaphylactoid purpura with intestinal perforation: report of a case and review of the Japanese literature. Pathol Int. 1994;44: 303–308. 8044297 10.1111/j.1440-1827.1994.tb03368.x

[16] Banerjee B, Rashid S, Singh E, Moore J. Endoscopic findings in Henoch-Schonlein purpura. Gastrointest Endosc. 1991;37: 569–571. 1936842 10.1016/s0016-5107(91)70835-3

[17] Yoshikawa N, Yamamura F, Akita Y, Sato T, Mitamura K. Gastrointestinal lesions in an adult patient with Henoch-Schonlein purpura. Hepatogastroenterology. 1999;46: 2823–2824. 10576353

[18] Esaki M, Matsumoto T, Nakamura S, Kawasaki M, Iwai K, Hirakawa K, et al GI involvement in Henoch-Schonlein purpura. Gastrointest Endosc. 2002;56: 920–923. 10.1067/mge.2002.129592 12447314

[19] Kim KY, Jang JY, Kim JW, Shim JJ, Lee CK, Dong SH, et al Acid suppression therapy as a risk factor for Candida esophagitis. Dig Dis Sci. 2013;58: 1282–1286. 10.1007/s10620-012-2520-x 23306845

[20] Kirkpatrick CH. Host factors in defense against fungal infections. Am J Med. 1984;77: 1–12. 6388323

[21] Ashman RB, Farah CS, Wanasaengsakul S, Hu Y, Pang G, Clancy RL. Innate versus adaptive immunity in Candida albicans infection. Immunol Cell Biol. 2004;82: 196–204. 10.1046/j.0818-9641.2004.01217.x 15061774

[22] Cho BA, Ko Y, Kim YS, Kim S, Choi MS, Kim IS, et al Phenotypic characterization of peripheral T cells and their dynamics in scrub typhus patients. PLoS Negl Trop Dis. 2012;6: e1789 10.1371/journal.pntd.0001789 22905277 PMC3419201

